# Application of Conservative Methods Based on Exercise in Reducing the Kyphotic Angle: A Meta-Analysis

**DOI:** 10.3390/healthcare13141742

**Published:** 2025-07-18

**Authors:** Vanja Dimitrijević, Bojan Rašković, Miroslav P. Popović, Dragan Marinković, Miloš Kojić, Siniša Nikolić, Nikola Jevtić, Borislav Obradović

**Affiliations:** 1 Faculty of Sports and Physical Education, University of Novi Sad, 21000 Novi Sad, Serbia; dimitrijevicvanja@gmail.com (V.D.); marinkovic@uns.ac.rs (D.M.); milos.kojic123@gmail.com (M.K.); boriscons@yahoo.com (B.O.); 2 Functionally Aware Motoric Activity (FAMA) Center, 21000 Novi Sad, Serbia; 3 Performance Zone, 21000 Novi Sad, Serbia; 4 Technical Faculty, Singidunum University, 11000 Belgrade, Serbia; miroslav.popovic@singidunum.ac.rs; 5 Institute of Physical Medicine, Rehabilitation and Orthopedic Surgery “Dr Miroslav Zotović”, 78000 Banja Luka, Bosnia and Herzegovina; sinisamnikolicbl@gmail.com; 6 Department of Physiotherapy, Faculty of Medicine, University of Banja Luka, 78000 Banja Luka, Bosnia and Herzegovina; 7 Scolio Centar, 21000 Novi Sad, Serbia; njevticns@gmail.com

**Keywords:** kyphotic angle, kyphosis, conservative methods, meta-analysis

## Abstract

**Background:** The aim of this research was to evaluate the size of the effect of conservative methods based on exercises on the reduction in the kyphotic angle through a meta-analysis. **Methods:** In our research, we followed the Cochrane guidelines and the PRISMA guidelines. In October 2024, we searched the following databases: Scopus, Pub Med, Web of Science, and Cochrane Library. The following keywords were used for the search: (“Kyphosis” OR “Hyperkyphosis” OR “Kyphotic angle” OR “Spinal curve” OR “Sagittal spinal”) AND (“Conservative methods” OR “Conservative treatment” OR “Corrective exercise” OR “Physical exercise” OR “Exercise therapy” OR “Training”). The risk of bias was assessed for each randomized trial using the Cochrane Risk of Bias Tool and the Methodological Index for Non-Randomized Studies (MINORS). The study’s main outcome and estimated effects were the kyphotic angle. R 4.3.2 software with the meta package was used for analyses, and standardized mean difference (SMD) and 95% confidence interval (CI), a randomized model for continuous outcomes, were used as effect size. **Results:** Twenty-one studies were included in the qualitative analysis, while 19 studies were included in the quantitative analysis. Depending on the analysis, the effect sizes ranged from (SMD = 0.27 to SMD = 0.73). Heterogeneity ranged from 0% to 58%, while the quality of evidence (GRADE) ranged from very low to moderate. **Conclusions**: Our meta-analysis indicates the positive effects of applying conservative methods based on exercise in subjects without and with osteoporosis diagnosed with hyperkyphosis.

## 1. Introduction

Kyphosis is a deformation of the spine with excessive backward curvature of the thoracic spine [1]. Kyphosis refers to the normal, physiological sagittal curvature of the thoracic spine, typically ranging from 20° to 45°. When this curvature exceeds 40° to 45°, particularly when accompanied by clinical symptoms such as postural deformity, pain, functional limitations, or aesthetic consequences, the condition is classified as pathological thoracic hyperkyphosis [2,3,4,5]. Thoracic hyperkyphosis has an incidence that ranges from 20% to 40% in the population [6]. Thoracic hyperkyphosis is more common in males than females and progresses during the adolescent growth spurt in girls while diminishing after the growth spurt [7]. The causes of hyperkyphosis can be various traumas and developmental anomalies, genetic links, osteoporosis, degenerative disc, inflammatory diseases, infectious diseases, age-related, and often the cause is unknown [3,4,6,8]. Treatment of osteoporosis may have limited benefits for the prevention of hyperkyphosis [6]. Given the degree of kyphosis, it is divided into two types: low-grade (such as the postural round back) and high-grade (angular gybes deformity, congenital kyphosis, Pott’s disease, and the most famous form, which is Scheuerman) [7]. Hyperkyphosis can also develop due to muscular and neuromuscular diseases. Hyperkyphosis can affect muscle efficiency; furthermore, this is considered an etiological factor in musculoskeletal conditions [9]. An increase in the degree of curvature can lead to breathing difficulties, limited range of motion, and cosmetic deformities [10,11,12].

Currently, various treatments are used for hyperkyphosis, including exercise therapy, taping, bracing, manual therapy, and surgery [13,14,15,16,17]. Wearing a brace is suggested for patients with 55 degrees of thoracic kyphosis or 40 degrees of thoracolumbar kyphosis until they reach adulthood; while wearing a brace does not affect patients with Riser’s sign 4 or 5 [7]. Braces should be used for more than 20 h a day. Wearing a brace should be used for at least 18 months [7]. Exercise therapy is one of the most common methods for managing hyperkyphosis [5]. Corrective exercise has been reported to be a safe and effective method for improving spinal deformity [9]. Some studies have shown that therapeutic exercises, especially those that increase the strength of the back extensor muscles, can reduce thoracic hyperkyphosis [9]. Also, exercises that affect the mobility of the spine [18], better activation, and better postural alignment [19,20,21] are recommended in the treatment of kyphosis and hyperkyphosis.

So far, four meta-analyses have been performed that examined the effect of exercise on the reduction in hyperkyphosis. A study by [22] reports that exercise has a positive effect on the reduction in thoracic hyperkyphosis but does not evaluate the effect on age groups or the effect of different treatment durations. A study by [23] also reports on the positive impact of exercises on reducing hyperkyphosis, with the fact that it brings the effects of the action on older and younger groups of subjects with different durations of treatment. In addition, it reports on the effects of applying bracing on subjects with hyperkyphosis. The study [24] concludes that interventions aimed at hyperkyphosis have a positive effect on the back extensor muscles in the elderly. The study by [25] reported moderate effects of corrective exercises in reducing hyperkyphosis in three age groups: Older, Middle, and Younger. Although several meta-analyses have previously explored the effects of exercise on thoracic hyperkyphosis, these studies were limited in scope and methodology. Most included a smaller number of trials, lacked rigorous subgroup analyses, and did not assess the influence of study quality, age distribution, treatment duration, or the presence of osteoporosis on treatment outcomes. Therefore, our meta-analysis aimed to update and expand the existing evidence base by including a larger number of studies, applying GRADE quality assessments, and performing detailed subgroup analyses (e.g., by age, osteoporosis status, and intervention duration). These additions allow for a more comprehensive understanding of the effectiveness and limitations of conservative exercise-based treatments for hyperkyphosis across diverse populations. In our study, hyperkyphosis is addressed as a clinical condition, independent of its underlying etiology.

This meta-analysis of ours aims to describe which conservative exercise-based methods are used in the reduction in hyperkyphosis, to try to differentiate the effect on different age groups, to compare the effects of different durations of treatment, to examine whether the application of exercises affects the reduction in hyperkyphosis in subjects with osteoporosis, and to investigate whether the quality of the included studies affects the overall synthesis of the obtained results.

## 2. Materials and Methods

### 2.1. Study Design

The Cochrane Collaboration guide [26] and the PRISMA guidelines [27] were used to create this meta-analysis of ours, and it was registered in the PROSPERO database (CRD42024502701).

### 2.2. Data Sources, Search Strategy, and Selection Process

**Figure 1:**
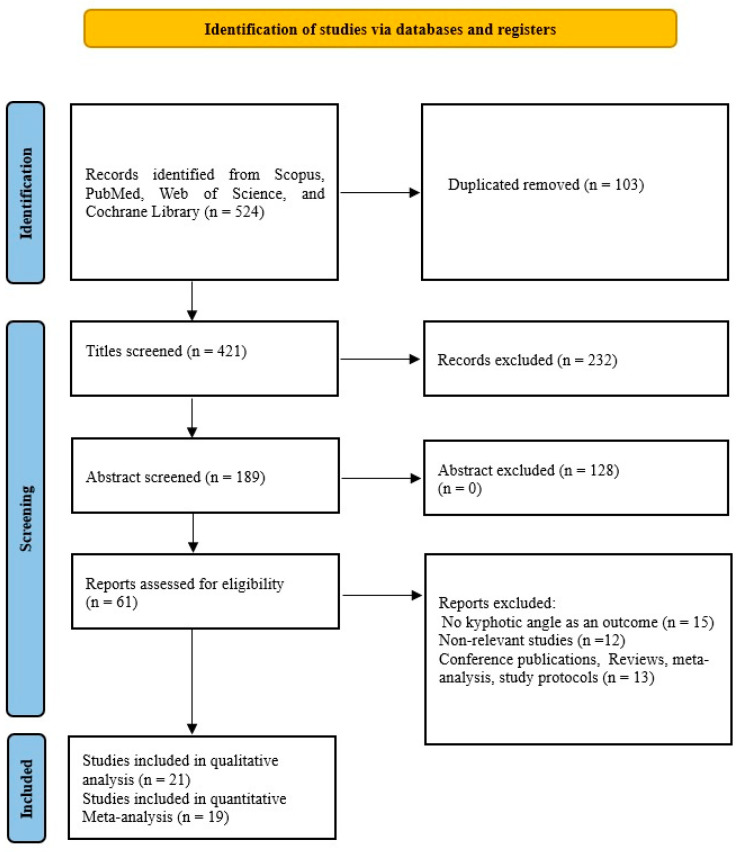
PRISMA flow diagram of study selection and inclusion/exclusion process.

Our search was developed to find reference studies that investigated the effects of different conservative exercise-based methods on subjects with kyphosis and hyperkyphosis. PubMed, Cochrane Library, Scopus, and Web of Science are the databases we searched in October 2024. In our search, we used the following keywords: (“Kyphosis” OR “Hyperkyphosis” OR “Kyphotic angle” OR “Spinal curve” OR “Sagittal spinal”), AND (“Conservative methods” OR “Conservative treatment” OR “Corrective exercise” OR “Physical exercise” OR “Exercise therapy” OR “Training”). Figure 1 shows our detailed search.

For inclusion and exclusion of studies, we followed the PICOS criteria described in PRISMA [27].

Inclusion criteria: P (population): subjects of any age with increased thoracic kyphotic curvature, including both clinically diagnosed hyperkyphosis, where at least one group had a mean value over 40 degrees, I (intervention): conservative exercise-based methods, C (comparison): the control group either received therapy with conservative exercise-based methods; or did not receive any treatment, or they received home exercises. O (outcome): Kyphotic angle, S (study design): only interventional comparative studies (randomized and non-randomized controlled studies) were included to allow for effect size calculation and minimize bias that were published from 2000 to the date of the search, without language restrictions. Exclusion criteria: programs based on the use of braces, breathing exercises, conference publications, study protocols, meta-analyses, book reviews, books, and systematic reviews. Single-arm or uncontrolled studies were excluded. Two investigators (B.R. and B.O.) performed the selection for inclusion or exclusion of studies, and any disagreements were resolved by consultation and consensus. After that, they performed data extraction independently. The table included the following variables: first author, year of publication, program type, number of participants, assessments of kyphotic angle, age, exercise per week, and duration.

### 2.3. Quality Assessment

Study quality was assessed by two investigators independently. To evaluate randomized studies, the Cochrane Risk of Bias Tool [28] was used, which assesses bias in seven sources. High, low, or unclear risk was determined for each study. MINORS was used for the quality of non-randomized studies [29]. MINORS includes 12 items. A score ≥14 was used to include non-randomized studies.

### 2.4. Statistical Analysis

Statistical analysis was performed using R 4.3.2 software with the meta package [30]. The effect size was calculated for the Kyphotic angle outcome. Standardized mean difference (SMD) and 95% confidence intervals (CI) were determined for continuous outcomes with a random model. Using Cohen’s guide, values of ≥0.2, ≥0.5, and ≥0.8 were defined as small, medium, and large, and *p* < 0.05 was considered statistically significant [31]. After the basic analysis, subgroup analyses were performed for the factors of age, duration of treatment, risk of bias, and, in the older group, the factor of Osteoporosis. Heterogeneity was assessed by Higgins’s I^2^ test and *p*-value [32]. Publication bias was examined with Egger’s test [33].

### 2.5. Quality of Evidence

The quality of meta-analysis records was assessed using the Grading of Recommendations, Assessment, Development, and Evaluation (GRADE) system tool [34]. The quality of the evidence was rated as “High”, “Moderate”, “Low”, and “Very-Low”, with certainty that the true effect size is close to the obtained effect size. The reasons for reducing the quality of records were: the risk of bias, Inconsistency, Indirectness, Imprecision, and Publication bias. Due to the risk of bias, the quality is downgraded by one level if the probable bias would seriously change the results, when most studies come from low and moderate risk of bias. The overall risk of bias for each study was assessed as “Low”, “Moderate”, or “High”. When the proportion of information from high-risk studies is sufficient for interpretation, and there is a serious risk of bias, downgrade by one level, and for a very serious risk, by two levels. Inconsistency is minimized if there is a large variation in effect size across studies, if there is little or no overlap in the confidence intervals associated with the effect, and if statistical tests show large heterogeneity. Indirectness was reduced if included studies had limitations regarding population, intervention, comparator, and outcomes. Imprecision was decreased if the total number of subjects was less than 400 or if the lower or upper confidence limit was greater than 0.5 of the SMD in both directions. Publication bias, if there was an asymmetry of the meta-analysis funnel with at least 10 included studies, underreporting of negative results, and if conflicts of interest of researchers were reported [35].

## 3. Results

### 3.1. Study Selection and Characteristics

From a total of 524 publications found through four databases, we included 21 studies in our qualitative analysis and 19 in our quantitative analysis. The characteristics of the included studies are shown in Table 1. Of the total studies, 17 were randomized, while 4 were non-randomized. Studies [36,37] that were randomized were not included in the quantitative meta-analysis because their results were influenced by increased heterogeneity and were therefore excluded. There were a total of 1207 respondents in the qualitative synthesis, while there were 1084 respondents in the quantitative synthesis. Of the total number of respondents in the quantitative synthesis, 577 (53.22%) were in the experimental group, while 507 (46.78%) were in the control group. The studies [38,39] had two experimental groups with different types of treatment and one control group and thus were included in the meta-analysis, so there were 21 comparisons. In the experimental group, the mean sample size was 27.58 ± 16.98 (range 11–81), and in the control group, it was 26.68 ± 16.98 (range 9–83). The mean duration of treatment was 14.53 ± 11.58 weeks (range 4–48). The size of the kyphotic curve of the spine is determined by the kyphotic angle. This deformity is diagnosed by spinal radiography [40,41,42,43], spinal mouse device [14,44,45,46], kyphometer [40,41,42,43,44,45,46,47], inclinometer [15,38,39,48,49,50], 4D formetric device [36], computerized bio-photogrammetry [51], six-camera motion analysis system [9], or flexicurve ruler [5,37,52]. Studies [41,42,43] using radiography measured the angle of thoracic kyphosis with the Cobb angle. All studies used strengthening and/or stretching exercises as therapy. In addition to exercises, the study [14] used manual mobilization and taping, the study [15] in addition to the exercises only manual therapy, while the study [48] in addition to the exercises used kinesiotaping. The study [41] uses the Schroth method, the study [47] uses Yoga exercises, and the study [51] uses Pilates, while the other studies use different variations of corrective exercises, as shown in Table 1. Four studies come from Iran [9,37,45,52] 3 from Turkey [39,46,48] 3 from Australia [15,38,43] 3 from the United States of America [5,40,47] 2 from Egypt [36,42] 2 from South Korea [49,50] and one each from China [44], Brazil [51], Belgium [14], and Israel [41].

### 3.2. Risk of Bias

**Figure 2:**
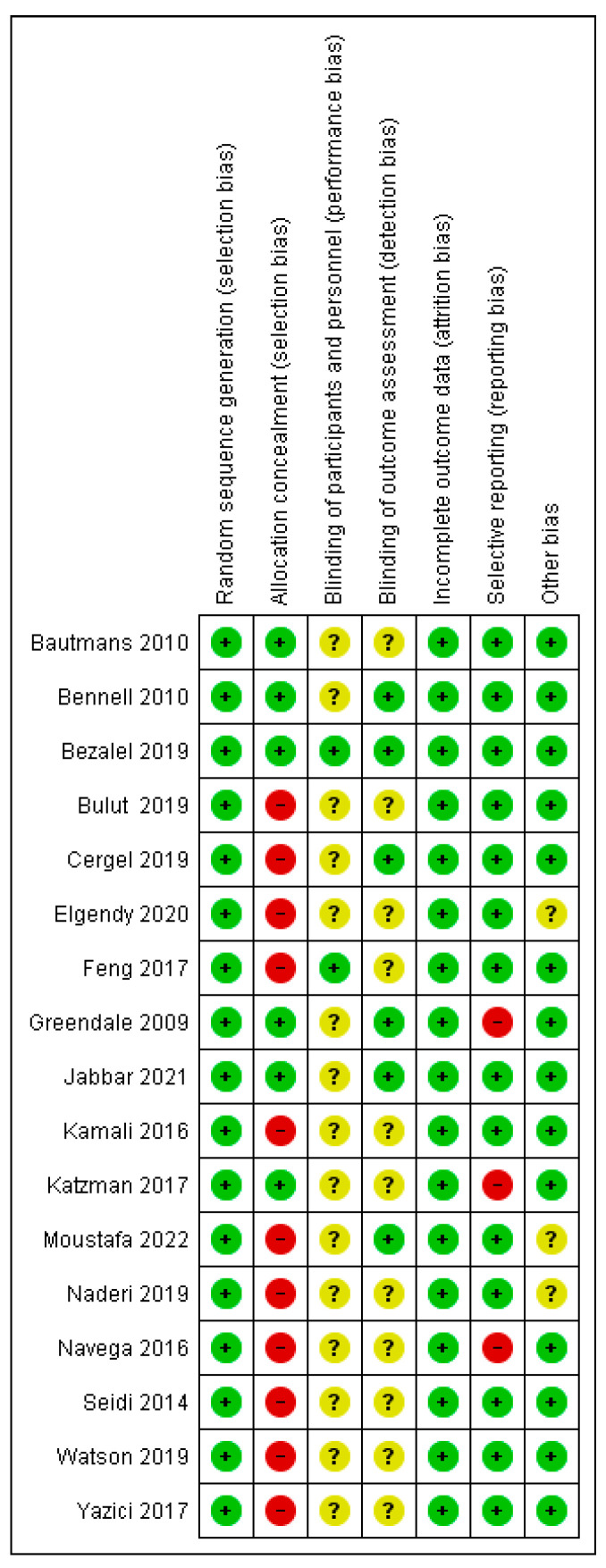
Risk of bias for randomized studies [9,14,15,36,37,39,40,41,42,43,44,45,46,47,48,51,52].

**Figure 3:**
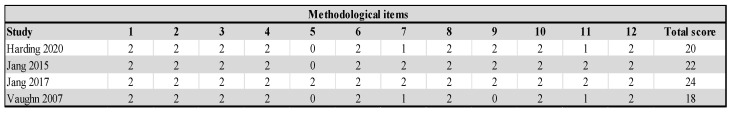
Risk of bias for non-randomized studies [5,38,49,50].

Of the 17 randomized studies, 7 were assessed as low risk, 9 as moderate, including studies that were not included in the meta-analysis, and one as high risk. Out of a total of 119 items, 73 (61.34%) were low risk, 32 (26.89%) unclear risk, and 14 (11.76%) high risk. Eleven studies [9,36,37,39,42,43,44,46,48,51,52] had a high risk in the “allocation concealment” item, while three studies [40,47,51] had “selective reporting”, and because of that, they had a high risk. Due to the very nature of the treatment, the patients were not blinded, although two studies report blinding in this item as well (Figure 2). All non-randomized studies [5,38,49,50] were comparative, with the lowest score of 18 and the highest score of 24, the maximum score in the MINORS tools (Figure 3). In the subgroup analysis, the influence of the study quality on the effect size was examined. Studies are classified as low risk, moderate risk, and high risk. All non-randomized studies were classified as high-risk.

### 3.3. Meta-Analysis

#### 3.3.1. The Overall Effect of Conservative Exercise-Based Methods

**Figure 4:**
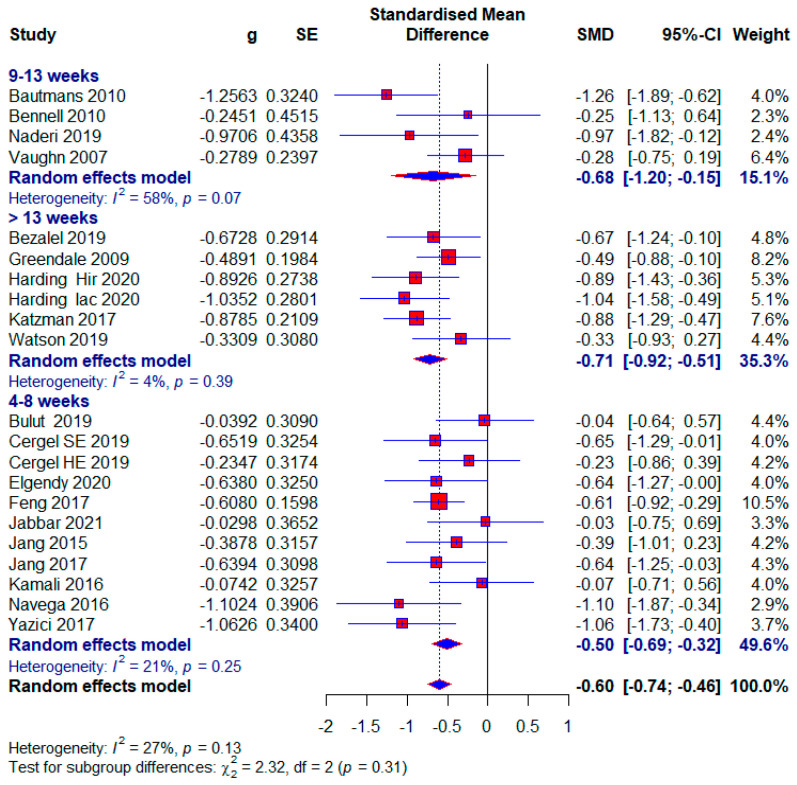
Forest plot—Duration: Hir—high-intensity progressive resistance and impact training; Iac—isometric axial compression; SE—strength exercises; HE—home-based exercise [5,9,14,15,38,39,40,41,42,43,44,45,46,47,48,49,50,51,52].

**Figure 5:**
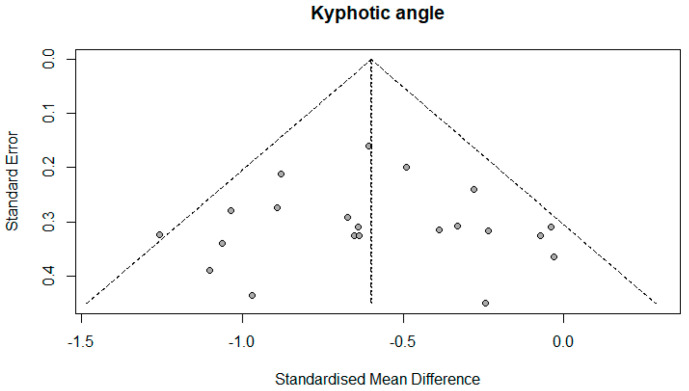
Funnel plot.

The number of studies that measured the kyphotic angle was 19. There were a total of 1084 subjects, of which 577 (53.22%) were in the experimental group and 507 (46.78%) in the control group. Ten studies (52.63%) in the control group did not have any treatment; that is, 272 (25.09%) respondents out of the total number. The performed analysis shows statistically significant results with a moderate effect size (SMD = −0.60; 95% CI = −0.74, −0.46; *p* < 0.0001; GRADE: low (Table 2) and heterogeneity (I^2^ = 27%, *p* = 0.13) (Figure 4). Sensitivity analysis shows that excluding the study 15 would reduce the heterogeneity to 18% and the effect size to (SMD = −0.57). Publication bias was not statistically significant by Egger’s test (intercept 0.103; 95% CI = −1.868, 2.075; *p* = 0.92) (Figure 5).

#### 3.3.2. The Influence of the Duration of the Treatment on the Effect Size

- Short duration of treatment

We classified the shortest treatment into the subgroup between 4 and 8 weeks. Ten studies used this duration of treatment, and a total of 551 (48.99%) subjects, of which 61 (11.49%) subjects in the control group did not receive any treatment. The results of the meta-analysis show statistical significance with a moderate effect size (SMD = −0.50; 95% CI = −0.69, −0.32; *p* < 0.0001; GRADE: moderate (Table 2) and heterogeneity (I^2^ = 21%, *p* = 0.25) (Figure 4).

- The median duration of treatment

The median duration of treatment included studies between 9 and 13 weeks. Four studies used this duration of treatment, and a total of 163 (15.03%) subjects were included. After the meta-analysis, statistical significance was achieved with a moderate effect size (SMD = −0.68; 95% CI = −1.20, −0.15; *p* = 0.001; GRADE: low (Table 2) and heterogeneity (I^2^ = 58%, *p* = 0.07) (Figure 4).

- Maximum duration of treatment

In this subgroup, we included studies that had more than 13 weeks of treatment. Five studies were included in this subgroup, and a total of 416 (38.38%) subjects participated, and 124 (29.8%) of them did not receive any treatment in the control group. This subgroup showed the largest effect size, which was still moderate (SMD = −0.71; 95% CI = −0.92, −0.51; *p* < 0.0001; GRADE: moderate (Table 2) and heterogeneity (I^2^ = 4%, *p* = 0.39) (Figure 4).

#### 3.3.3. The Influence of Conservative Exercise-Based Methods on Different Age Groups

- Younger age group

**Figure 6:**
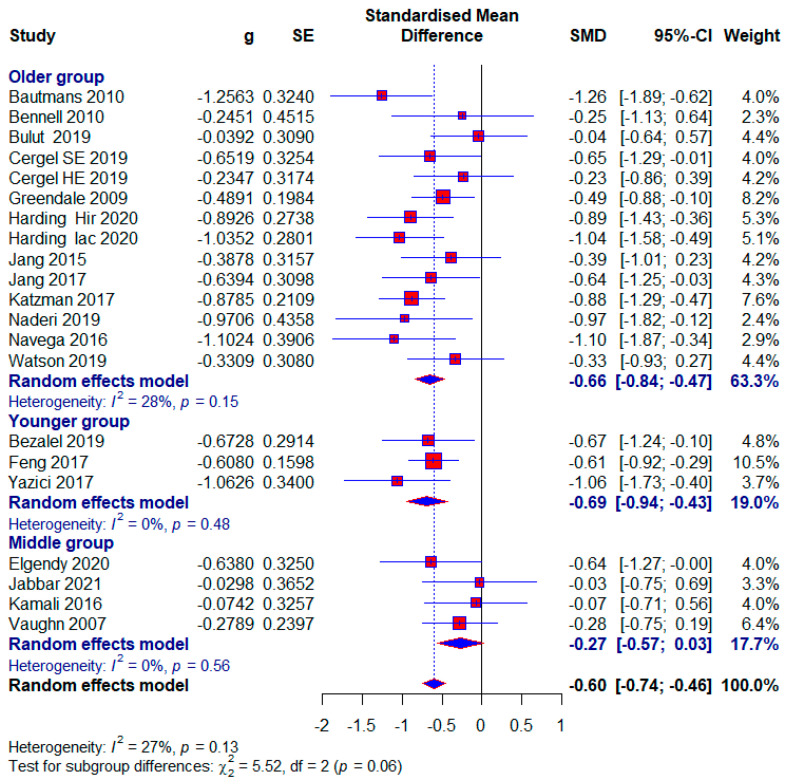
Forest plot—Age: Hir—high-intensity progressive resistance and impact training; Iac—isometric axial compression; SE—strength exercises; HE—home-based exercise [5,9,14,15,38,39,40,41,42,43,44,45,46,47,48,49,50,51].

Three studies were classified in the younger group, with 254 (23.43%) subjects, and 20 (7.87%) of them in the control group did not receive any treatment. The group ranged in age from 10 to 20 years old. Statistical significance with a moderate effect size appears in this subgroup (SMD = −0.69; 95% CI = −0.94, −0.43; *p* < 0.0001; GRADE: low (Table 2) and heterogeneity (I^2^ = 31%, *p* = 0.23) (Figure 6).

- Middle age group

Four studies were included in the middle group, with 180 (16.61%) respondents, of which 39 (21.67%) in the control group did not receive any treatment. We formed the middle age group because study 45 included subjects aged 24.25 ± 2.42 and 23.85 ± 2.56 years, in the study [45] subjects with 21.66 ± 2.05 and 19.73 ± 5.81 years, in the study19 with 23.1 ± 2.3 and 23.6 ± 2.9. years, and in the study5 with 39.8 ± 13.2 years. A small effect size with statistical significance appears in this subgroup (SMD = −0.27; 95% CI = −0.57, 0.03; *p* < 0.0001; GRADE: very-low (Table 2) and heterogeneity (I^2^ = 0%, *p* = 0.56) (Figure 6).

- Older age group

Twelve studies were included in the Older group, with 696 (64.21%) respondents, of which 151 (21.70%) in the control group did not receive any treatment. Statistical significance with a moderate effect occurs in this subgroup (SMD = −0.66; 95% CI = −0.84, −0.47; *p* < 0.0001; GRADE: low (Table 2) and heterogeneity (I^2^ = 28%, *p* = 0.15) (Figure 6). Excluding study [14] would reduce the effect size to 0.62 and the heterogeneity to 17%.

#### 3.3.4. The Influence of Study Quality on Effect Size

- Low risk of bias

**Figure 7:**
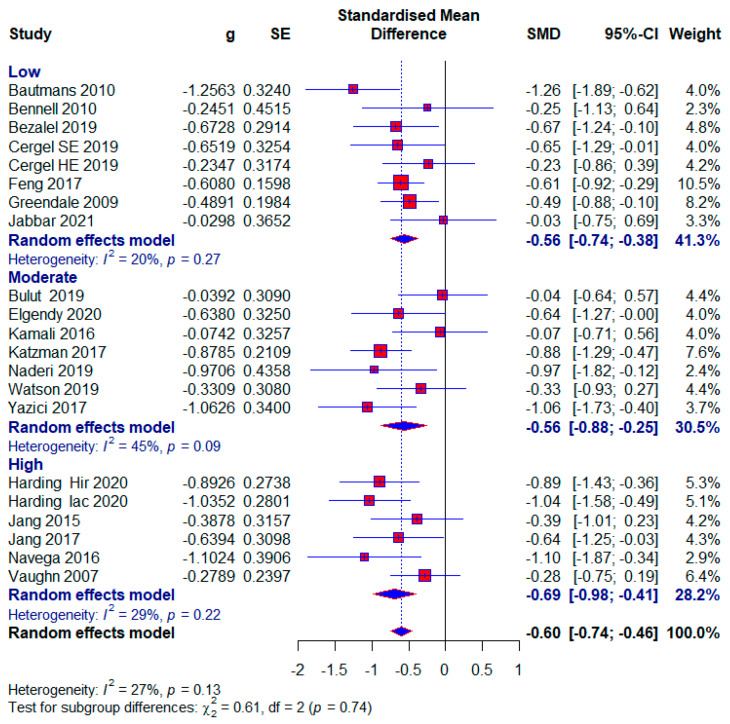
Forest plot—Risk of bias: Hir—high-intensity progressive resistance and impact training; Iac—isometric axial compression; SE—strength exercises; HE—home-based exercise [5,9,14,15,38,39,40,41,42,43,44,45,46,47,48,49,50,51,52].

Seven studies were assessed as being at low risk of bias, with 477 (44%) subjects. A moderate effect size with statistical significance appears in this subgroup (SMD = −0.56; 95% CI = −0.74, −0.38; *p* < 0.0001; GRADE: moderate (Table 2) and heterogeneity (I^2^ = 20%, *p* = 0.27) (Figure 7).

- Moderate risk of bias

Also, seven studies were examined with a moderate risk of bias, with 327 (30.17%) respondents. The same effect size appears as in the low-risk-of-bias group with also statistical significance (SMD = −0.56; 95% CI = −0.88, −0.25; *p* < 0.0001; GRADE: low (Table 2) and heterogeneity (I^2^ = 45%, *p* = 0.09) (Figure 7).

- High risk of bias

**Figure 8:**
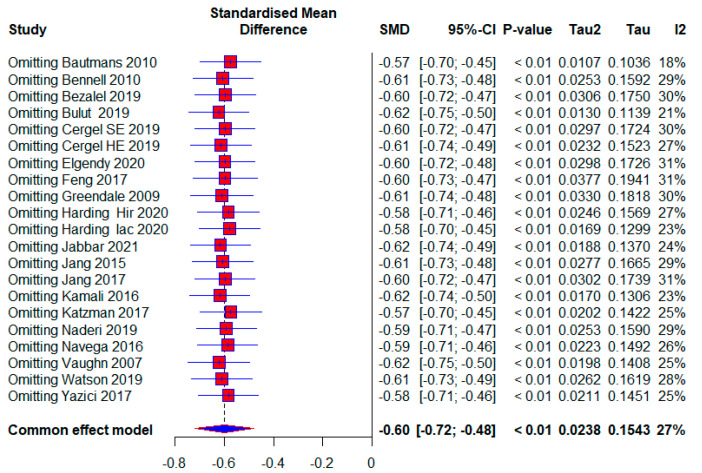
Leave-one-out: Hir—high-intensity progressive resistance and impact training; Iac—isometric axial compression; SE—strength exercises; HE—home-based exercise [5,9,14,15,38,39,40,41,42,43,44,45,46,47,48,49,50,51,52].

Five included studies were assessed as having a high risk of bias, with 280 (25.83%) respondents. A moderate effect size with statistical significance also appears in this subgroup (SMD = −0.69; 95% CI = −0.98, −0.41; *p* < 0.0001; GRADE: very low) (Table 2) and heterogeneity (I^2^ = 29%, *p* = 0.13) (Figure 7). The overall effect size would vary from 0.57 to 0.62; if one of the studies were excluded [5,9,45,48] (Figure 8). 

#### 3.3.5. The Effect of Conservative Exercise-Based Methods on Subjects with Osteoporosis

- Osteoporotic subjects

**Figure 9:**
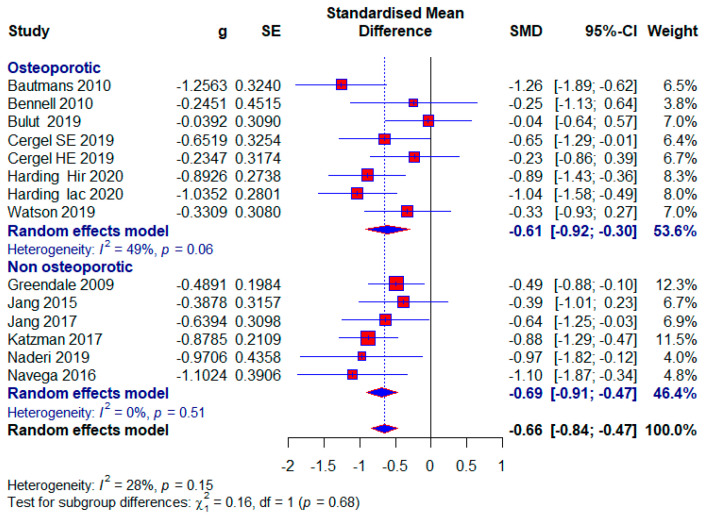
Forest plot—Osteoporosis: Hir—high-intensity progressive resistance and impact training; Iac—isometric axial compression; SE—strength exercises; HE—home-based exercise [14,15,38,39,40,43,47,48,49,50,51,52].

Seven included studies conducted research on osteoporotic subjects, with 332 (30.63%) subjects, of which 65 (19.58%) in the control group did not receive any treatment. The application of treatment to osteoporotic subjects had moderate effects with the appearance of statistical significance (SMD = −0.61; 95% CI = −0.92, −0.30; *p* < 0.0001; GRADE: very low (Table 2) and heterogeneity (I^2^ = 49%, *p* = 0.06) (Figure 9).

- Subjects without osteoporosis

Six included studies conducted research with subjects who did not have osteoporosis, and there were a total of 344 (31.73%) subjects, of which 126 (36.63%) in the control group did not receive any treatment. A moderate effect size with statistical significance also appears in this subgroup (SMD = −0.69; 95% CI = −0.91, −0.47; *p* < 0.0001; GRADE: very low (Table 2) and heterogeneity (I^2^ = 0%, *p* = 0.51) (Figure 9).

#### 3.3.6. Studies Without Treatment in the Control Group

**Figure 10:**
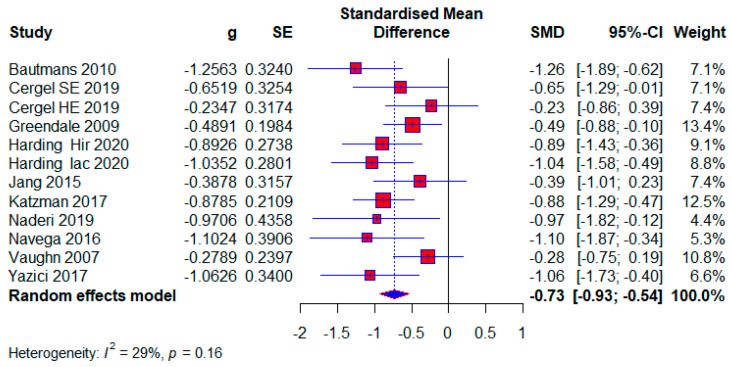
Forest plot—The Contol group has no treatment: Hir—high-intensity progressive resistance and impact training; Iac—isometric axial compression; SE—strength exercises; HE—home-based exercise [5,14,38,39,40,46,47,49,51,52].

Ten studies used no treatment in the control group. They had a total of 592 (54.61%) respondents, of which there were 320 (54.05%) in the experimental group and 272 (45.95%) in the control group. The effect size is still within the limits of moderate: (SMD = −0.73; 95% CI = −0.93, −0.54; *p* < 0.0001; GRADE: low (Table 2) and heterogeneity (I^2^ = 29%, *p* = 0.16) (Figure 10).

## 4. Discussion

### 4.1. Summary of Main Results

Our study aimed to use meta-analysis in research methodology to determine the effect size of different conservative exercise-based methods for reducing kyphotic angle in patients with kyphosis and hyperkyphosis. The quantitative analysis included 19 studies with a total of 1084 participants. Kyphotic angle was the outcome used to estimate the effect size. In the subgroup analysis, effect sizes were provided for factors such as treatment duration, age, study quality, and the presence or absence of osteoporosis, with effect sizes ranging from 0.27 to 0.71. The overall effect size for all included studies was (SMD = 0.60), while studies with no treatment in the control group tended to show a larger effect size (SMD = 0.73) (Figure 4, Figure 5, Figure 6, Figure 7, Figure 8, Figure 9 and Figure 10). The quality of evidence (GRADE) for all included studies was low, with variability depending on additional analyses ranging from very low to moderate.

### 4.2. Overall Completeness and Applicability of Evidence

In our study, patients were diagnosed with kyphosis and hyperkyphosis, and various therapies based on exercise were used. Since almost every included study had a different type of exercise treatment (Table 1), we were unable to differentiate according to the quality of individual treatments. Treatment durations ranged from four weeks to twelve months. Exercise therapy is effective for managing kyphosis and hyperkyphosis regardless of duration, with a consistent moderate effect size. The largest effect size is observed in the >13-week subgroup, with minimal heterogeneity, suggesting that longer interventions may provide more significant and reliable benefits. This aligns with the principle that sustained exercise programs can better influence posture and spinal health over time. The moderate effect size in the 9–13 weeks subgroup is accompanied by substantial heterogeneity (I^2^ = 58%). This variability may result from differences in study populations, exercise protocols, or adherence levels. Clinicians should carefully evaluate specific interventions in this duration subgroup for consistency. The results were followed by moderate-quality evidence. Moderate effectiveness is observed even with shorter interventions, which may be beneficial for patients with time or compliance constraints. However, longer programs are likely more impactful, as suggested by the higher effect sizes in the longer-duration group (Figure 4). Most participants were older adults, with a significantly smaller number being middle-aged and adolescents. Exercise therapy showed similar effects in the oldest and youngest patients, though slightly higher in the youngest, while middle-aged patients showed the least effects (Figure 6). The results show that; Conservative exercise therapies play a crucial role in managing kyphosis and hyperkyphosis across all age groups. When appropriately tailored, these interventions can reduce progression, improve physical function, and enhance overall well-being. Early intervention, consistency, and a multidisciplinary approach maximize outcomes and prevent long-term complications. The results in all three age groups are limited by the quality of the evidence, and the younger age group and middle-aged age group are limited by the small number of studies. The similar effect sizes across studies with low, moderate, and high risks of bias suggest that the overall efficacy of conservative exercise therapy is robust, regardless of the quality of the included studies. This consistency strengthens the confidence in the positive effect of exercise therapy for managing kyphosis and hyperkyphosis. Studies with a high risk of bias show slightly larger effect sizes, which might reflect overestimation due to methodological limitations. This emphasizes the need for interpreting findings from high-risk studies with caution. Low-risk studies demonstrate a moderate effect size with low heterogeneity, making their findings more reliable for clinical decision-making. Conservative exercise therapy appears effective across all quality levels, making it a valuable intervention for kyphosis and hyperkyphosis management. Clinicians can use these findings to recommend exercise therapy with confidence while understanding that high-quality studies provide the most dependable evidence (Figure 7). Exercise therapy has equally good effects in patients with osteoporosis and those without osteoporosis; however, the results of patients without osteoporosis are somewhat better. This highlights the versatility of exercise therapy in managing kyphosis and hyperkyphosis, irrespective of underlying bone health status. While effective, the slightly higher heterogeneity in the osteoporotic group suggests that individual patient factors (e.g., severity of osteoporosis) may influence outcomes and require tailored approaches. With no observed heterogeneity and a strong moderate effect size, exercise therapy appears consistently effective for non-osteoporotic individuals, potentially serving as a first-line treatment for postural improvement. These results contradict the claims made by the studies [6]. And these results in both subgroups are limited by very low quality of evidence. Sensitivity analysis excluding one study showed that results varied from (SMD = 0.57) to (SMD = 0.62), with heterogeneity ranging from (I^2^ = 18%) to (I^2^ = 31%) (Figure 8).

### 4.3. Quality of the Evidence

Only three records were supported by moderate-quality evidence (GRADE), while six records were supported by low-quality evidence, and four records had very low-quality evidence. Four studies were non-randomized, while the remaining 15 studies in the quantitative analysis were randomized. Of the randomized studies, only study [51] had a high risk in two items. The item “allocation concealment” most frequently showed high risk, while three studies had high risk in the item “selective reporting”, which reduces the methodological quality of the included randomized studies. All non-randomized studies were comparative with satisfactory test results and were included in the study, but they were categorized as high-risk. Heterogeneity ranged from 0% to 58%, depending on the subgroup. Studies with results leading to heterogeneity were immediately excluded from further analysis. Studies [40,47,51] did not present results in the manner required for meta-analysis, and these studies were rated as high risk in the “selective reporting” item. This issue was addressed according to instructions [28,53,54]. The publication bias of the included studies was not statistically significant. The search for studies was comprehensive, using four databases without language restrictions. We believe that there are no studies that meet the criteria and were not included in our analysis.

### 4.4. Comparison with Results of Other Meta-Analyses

We found four meta-analyses addressing the same issue, published in 2019 [22] and 2021 [23,24,25]. Study [22] reports a nearly moderate effect size (SMD = 0.49; k = 8) with heterogeneity (I^2^ = 67%) for the application of therapy on the kyphotic angle, combining results from all studies. Study [24] yields results (SMD = 0.31; k = 9) with heterogeneity (I^2^ = 77%) for the same item. Our previous study [25] with the same number of included studies as the ones mentioned earlier yielded results (SMD = 0.50; k = 9) with heterogeneity (I^2^ = 24%). Our current study indicated that a larger number of included studies led to an increased effect size and greater homogeneity compared to studies [22,24], while compared to our previous research, there was an increase in effect size but less disruption inhomogeneity. Results from our previous research [25] showed that the youngest participants experienced moderate benefits, the middle-aged group demonstrated smaller improvements, and the oldest participants had the most pronounced effects, despite some heterogeneity in consistency across studies. In our current study, the youngest group exhibited greater improvements compared to previous findings, while the middle-aged group showed similar results with minimal variation. The oldest participants continued to show significant benefits, with reduced heterogeneity across studies compared with our earlier research. Study [23] shows that treatments shorter than three months have the following values: (SMD = 2.81; k = 6) with heterogeneity (I^2^ = 97%), making these results invalid due to high heterogeneity. Such treatments for older adults have values: (SMD = 0.30; k = 4) with heterogeneity (I^2^ = 52%). Treatments longer than three months for older adults have the following values: (SMD = 0.19; k = 3) with heterogeneity (I^2^ = 60%). Unlike previous meta-analyses, only our current study examines the impact of study quality on effect size and the impact of treatment application on participants with or without osteoporosis. In addition, our study applies more refined subgroup categorizations (e.g., younger vs. middle-aged vs. older adults), stratifies outcomes by treatment duration, and explicitly evaluates the GRADE certainty of the evidence. These methodological enhancements were lacking in prior meta-analyses, which often presented pooled estimates with considerable heterogeneity and limited interpretability. Therefore, our study aims to address both clinical and methodological gaps in the current literature. Among all the mentioned meta-analyses, our current study includes the largest number of studies, and thus the results obtained in the research carry more weight.

### 4.5. Limitations and Recommendations

This study has several important limitations: First, the main limitation is the large number of different types of exercise-based programs used in the included studies. This variation prevented us from methodologically differentiating and classifying the treatments according to their structure or quality. The methodological heterogeneity arising from this diversity—including differences in structure, components, and application methods—limited our ability to compare and synthesize the data in a standardized way. We recommend that future studies adopt clearer frameworks for describing and classifying exercise interventions to enhance consistency and enable more robust meta-analytic synthesis. Second, the wide age range of the included participants may have introduced clinical variability. Participants from younger populations may experience hyperkyphosis due to different etiological mechanisms compared to older populations (e.g., postural vs. osteoporotic causes). We addressed this issue by conducting subgroup analyses based on age. Third, the scope of our outcome assessment was limited to a single parameter—the thoracic kyphotic angle. Although this outcome was the most consistently reported across studies, it prevented us from analyzing the effects of exercise on broader health domains such as physical function, posture, pain, or quality of life. Future research should include a wider range of standardized and clinically meaningful outcome measures. Fourth, while we performed subgroup analyses based on intervention duration, other important program characteristics—such as intensity, frequency, and content—could not be reliably analyzed due to inconsistent reporting across studies. This is a methodological limitation that should be addressed in future trials by providing comprehensive and detailed intervention protocols. Fifth, although data on follow-up periods were extracted, they were often inconsistently reported and varied substantially in length. Consequently, our meta-analysis focused on immediate post-intervention outcomes rather than long-term follow-up effects. Future studies should include standardized and extended follow-up durations to evaluate the sustainability of intervention effects over time. Sixth, the inclusion of non-randomized studies in the meta-analysis may represent a limitation due to potential bias. However, we addressed this concern by conducting subgroup analyses based on study quality and risk of bias. Our GRADE assessment also reflects this issue and was used to guide the strength of our conclusions. The main limitation of this study is the large number of different types of programs used in treatment, which prevented us from methodologically differentiating and classifying the treatments according to their quality. Another important limitation is the methodological heterogeneity arising from the wide variation in exercise-based intervention programs across the included studies. These interventions differed in structure, components, and application methods, yet no unified classification was applied. This lack of systematic categorization limits the comparability of results across studies and may affect the overall interpretability of treatment effects. We recommend that future studies adopt clearer frameworks for describing and classifying exercise interventions to enhance consistency and enable more robust synthesis. Another limitation is the wide age range of the included participants. We included participants from younger populations in our meta-analysis, whose problems may have different etiologies compared to older populations. We addressed this issue by classifying participants into subgroups. A third limitation of our research is measuring only one outcome, the kyphotic angle, so the effects of exercise programs were not examined in a broader scope. Although the studies we included had a small number of common outcomes, apart from the kyphotic angle, which they assessed. Although duration was analyzed through subgroup comparisons, other intervention characteristics such as intensity, frequency, and content could not be reliably analyzed, and this is also a limitation. We recommend that future studies provide detailed intervention protocols. Although we extracted data on follow-up periods, these were often inconsistently reported and varied substantially. As a result, our analysis focused on post-intervention outcomes rather than long-term follow-up effects. Future studies should standardize and extend follow-up durations to assess sustained benefits of exercise interventions. Including non-randomized studies in the meta-analysis may also be a limitation, and we attempted to address this issue by analyzing subgroups based on the quality of the included studies. Based on the GRADE assessment, these are our basic recommendations:

### 4.6. For Patients

Younger and older age groups might experience moderate benefits from exercise programs targeting posture correction. However, the certainty of the evidence supporting these benefits is low to very low, so expectations should be cautious. Middle-aged patient evidence suggests limited effectiveness in this age group, with very low certainty. With or without osteoporosis patients, results are inconsistent, and the overall quality of evidence is very uncertain. Patients should consult with a healthcare provider to discuss whether an individualized exercise program is appropriate, taking into account their health status and goals. With or without osteoporosis patients, results are inconsistent, and the evidence is very uncertain. Consult with a specialist to tailor an exercise program that considers your bone health and overall fitness.

### 4.7. For Clinicians

Clinicians may consider implementing conservative exercise programs, especially for younger and older adults, as there is a trend toward benefit. However, due to the low to very low quality of evidence, any recommendation should be made with caution and tailored to the individual patient. For middle-aged patients, combining exercise with other interventions may be more appropriate given the less consistent outcomes. In osteoporotic patients, exercise may offer postural benefits, but safety and fracture risk should always be considered. Clinical decisions should be informed by patient characteristics, and expectations should be managed accordingly. When interpreting these findings, be mindful of the heterogeneity in treatment durations and patient populations. A personalized approach, regularly reassessing patient progress, might yield better outcomes.

### 4.8. For the Scientific Community

Future research should aim to reduce bias by prioritizing well-designed randomized controlled trials. There’s a need to explore standardized exercise protocols to address the heterogeneity seen in current studies. Investigating specific subgroups more deeply—considering age, bone density, and exercise type—could offer more precise insights. Long-term follow-up studies could provide better data on the sustained effects of exercise on kyphosis. Our main recommendations for future research on this topic would focus on standardizing other outcomes that could effectively evaluate changes resulting from various treatments in patients with kyphosis and hyperkyphosis. Given the very low to moderate GRADE ratings of current evidence, further studies are necessary to establish clearer clinical guidelines. Based on the results of meta-analyses published so far, such research shows that it deserves significant attention from the scientific community, and a standard could be established from the multitude of treatment protocols to be applied in future randomized studies.

## 5. Conclusions

Our study suggests that conservative exercise-based methods may have a moderate beneficial effect on reducing the thoracic kyphotic angle in individuals with kyphosis and hyperkyphosis. Effect sizes across subgroups ranged from small to moderate (SMD 0.27–0.71), indicating a consistent trend favoring exercise interventions. However, the certainty of the evidence—assessed using the GRADE framework—ranged from very low to moderate, primarily due to risk of bias, variability in treatment protocols, and small sample sizes in some studies. Therefore, while the findings are encouraging, they should be interpreted with caution. The results support the potential of exercise-based interventions as a non-invasive approach for managing postural and structural kyphosis, especially in younger and older adults. However, there is a clear need for future high-quality, randomized controlled trials with standardized interventions and longer follow-up periods to confirm and strengthen these conclusions. Our findings provide a foundation for clinical consideration of exercise therapy in kyphotic populations, but they should not yet be regarded as definitive evidence for clinical guidelines. In our opinion, our meta-analysis includes a sufficient number of studies and has conducted a sufficient number of subgroup analyses but lacks a sufficient number of outcomes. The mentioned limitations should serve as a basis for future research.

## Figures and Tables

**Table 1:** Basic characteristics of included studies.

Study	N	Program Type	Assessments of Kyphotic Angle	Kyphotic Angle (Degrees)	Age	Exercise per Week	Duration
Bautmans 2010 [14]	48	Manuel mobilization + exercises vs.	Spinal mouse device	>50°	69–83	18 sessions	3 months
		Control group (non treatment)					
Bennell 2010 [15]	20	Corrective exercises + manuel therapy vs.	Dualer Electric Inclinometer	59 ± 9	66.2 ± 8	(10× week)	10 weeks
		Control group (non treatment)		58 ± 12	68.3 ± 11.8		
Bezalel 2019 [41]	50	Schroth method vs.	Digital X-ray	>40°	10–17	daily	12 months
		Anti-gravitation exercises					
Bulut 2019 [48]	42	Functional correction technique + kinesiotaping	Inclinometer Dualer IQ	57.10 ± 10.30	63.1 ± 8.8	daily	6 weeks
		vs. Functional correction technique		55.95 ± 9.25	64 ± 7.08		
Cergel 2019 [39]	60	Back extensor strength training vs.	Dualer Digital Inclinometer	46.35 ± 8.79	58.90 ± 4.70	(3× week)	6 weeks
		Home-based exercise vs.		42.10 ± 7.15	60.20 ± 7.57		
		Control group (non treatment)		42.55 ± 6.68	59.65 ± 6.45		
Elgendy 2020 [42]	40	Manipulative therapy + Corrective	Digital X-ray	≥40°	24.25 ± 2.42	(3× week)	4 weeks
		exercises vs.			23.85 ± 2.56		
		Correctiveexercises					
Feng 2017 [44]	164	Corrective funktional exercises	Spinal mouse device	>40°	13–16	(2× week)	8 weeks
		vs. Exercise program					
Greendale 2009 [47]	105	Yoga vs.	Debrunner kyphometer	>40°	75.5 ± 7.4	(3× week)	6 months
		Control group (non treatment)					
Harding 2020 [38]	93	HiRIT vs.	Gravity-referenced	39.2 ± 9.0	61.9 ± 6.8	(2× week)	8 months
		IAC vs.	inclinometer (Plurimeter)	42.4 ± 11.5	70.3 ± 6.8		
		Control group (non treatment)					
Jabbar 2021 [45]	30	Corrective exercises (NASM) vs.	Spinal mouse device	49.26 ± 11.13	21.66 ± 2.05	(3× week)	8 weeks
		Sahrmann corrective exercises		50.4 ± 6.73	19.73 ± 5.81		
Jang 2015 [49]	41	Thorax correction exercises vs.	2 gravity-dependent inclinometer	57.2 ± 2.8	73.7 ± 5.6	(2× week)	8 weeks
		Home exercises		55.8 ± 4.7	76.4 ± 6.6		
Jang 2017 [50]	50	Corrective exercises vs.	Dual inclinometer	>40	>65	(2× week)	8 weeks
		Control group (education)					
Kamali 2016 [9]	39	Corrective exercise therapy	ProReflex, Qualysis	>45	23.1 ± 2.3	(3× week)	5 weeks
		vs. Manuel therapy			23.6 ± 2.9		
Katzman 2017 [40]	99	Strengthening exercise vs.	Debrunner kyphometer	56.8 ± 12.2	70.6 ± 0.6	(3× week)	6 months
		Control group (non treatment)		57.9 ± 12.9			
Moustafa 2022 [36]	80	Multimodal Program vs.	4D formetric device	82.15 ± 5.3	25.05 ± 3	(3× week)	10 weeks
		Strengthening exercise		83.15 ± 4.9	24 ± 4.2		
Naderi 2019 [52]	24	Corrective exercises vs.	Flexible ruler	>50	65–74	(3× week)	12 weeks
		Control group (non treatment)					
Navega 2016 [51]	31	Pilates vs.	Computerized	>40	67.71 ± 3.24	(2× week)	8 weeks
		Control group (non treatment)	bio-photogrammetry		65.4 ± 4.03		
Seidi 2014 [37]	37	Comprehensive corrective exercises	Flexicurve ruler	≥42	18–25	(3× week)	12 weeks
		vs. Control group (non treatment)					
Vaughn 2007 [5]	71	Home based exercises vs.	Flexicurve ruler	46.41 ± 8.71	39.8 ± 13.2	(4× week)	13 weeks
		Control group (non treatment)		44.63 ± 12.53			
Watson 2019 [43]	43	HiRIT vs.	DXA machine (Medix DR)	43.8 ± 9.3	64 ± 4	(2× week)	8 months
		Control group (home-based program)		35.4 ± 9.6	64 ± 5		
Yazici 2017 [46]	40	Corrective exercises vs.	Spinal mouse device	46.19 ± 3.29	16.84 ± 1.84	(3× week)	8 weeks
		Control group (non treatment)		45.71 ± 2.94	17.53 ± 2.07		

HiRIT: high-intensity progressive resistance and impact training; IAC: isometric axial compression; NASM: The National Academy of Sports Medicine.

**Table 2:** GRADE summary table. CI: confidence interval; SMD: standardized mean difference. Explanations: ^a^. Four of the included studies were non-randomized. Four non-randomized studies were considered high risk, while in the randomized studies, 61.34% of the items were low risk, 26.89% unclear risk, and 11.76% high risk. ^b^. Due to the large heterogeneity of the population in age. ^c^. Due to the heterogeneity of the population in age. ^d^. Two included studies had a high risk of bias. ^e^. The imprecision was reduced because the number of respondents was less than 400, and both the lower and upper confidence limits were greater than 0.5. ^f.^ The study Bezalel 2019 [41] examined a population aged 10 to 17. ^g^. Two studies used eight weeks of treatment, while one used 12 months of treatment. ^h^. The imprecision was reduced because the number of respondents was less than 400. ^i^. Two randomized studies were at high risk, and one study was non-randomized. ^j^. Heterogeneity in treatment duration. ^k^. The imprecision was reduced because the number of respondents was less than 400. ^l^. The high number of high-risk studies. ^m^. Heterogeneity in treatment duration. ^n^. Heterogeneity in the duration of treatment and heterogeneity in the age of the population. ^o^. Heterogeneity in treatment duration. ^p^. The imprecision was reduced because the number of respondents was less than 400. ^q^. All studies had high risk. ^r^. Heterogeneity in treatment duration. ^s^. The imprecision was reduced because the number of respondents was less than 400. ^t^. Three studies had high risk. ^u^. Heterogeneity in treatment duration. ^v^. The imprecision was reduced because the number of respondents was less than 400. ^w^. Three studies had high risk. ^x^. Heterogeneity in treatment duration. ^y^. The imprecision was reduced because the number of respondents was less than 400. ^z^. The high number of high-risk studies. ^aa^. Due to the large heterogeneity of the population in age.

Certainty Assessment							№ of Patients		Effect		Certainty	Importance
№ of Studies	Study Design	Risk of Bias	Inconsistency	Indirectness	Imprecision	Other Considerations	[Intervention]	[Comparison]	Relative (95% CI)	Absolute (95% CI)		
**Kyphotic angle all (follow-up: range 4 weeks to 48 weeks)**												
19	Randomised/non-randomised trials	serious ^a^	not serious	serious ^b^	not serious	none	577	507	-	SMD **0.6 SD lower** (0.74 lower to 0.46 lower)	**⊕⊕◯◯** **Low ^a,b^**	Important
**4–8 weeks (follow-up: range 4 weeks to 8 weeks)**												
10	Randomised/non-randomised trials	not serious	not serious	serious ^c^	not serious	none	275	276	-	SMD **0.5 SD lower** (0.69 lower to 0.32 lower)	**⊕⊕⊕◯** **Moderate ^c^**	Critical
**9–13 weeks (follow-up: range 9 weeks to 13 weeks)**												
4	Randomised/non-randomised trials	serious ^d^	not serious	not serious	serious ^e^	none	84	79	-	SMD **0.68 SD lower** (1.2 lower to 0.15 lower)	**⊕⊕◯◯** **Low ^d,e^**	Critical
**>13 weeks (follow-up: range 13 weeks to 48 weeks)**												
5	Randomised/non-randomised trials	not serious	not serious	serious ^f^	not serious	none	218	198	-	SMD **0.71 SD lower** (0.92 lower to 0.51 lower)	**⊕⊕⊕◯** **Moderate ^f^**	Critical
**Younger age group**												
3	Randomised trials	not serious	not serious	serious ^g^	serious ^h^	none	126	128	-	SMD **0.61 SD lower** (0.94 lower to 0.43 lower)	**⊕⊕◯◯** **Low ^g,h^**	Critical
**Middle age group**												
4	Randomised/non-randomised trials	serious ^i^	not serious	serious ^j^	serious ^k^	none	90	90	-	SMD **0.27 SD lower** (0.57 lower to 0.03 higher)	**⊕◯◯◯** **Very low ^i,j,k^**	Critical
**Older age group**												
12	Randomised/non-randomised trials	serious ^l^	not serious	serious ^m^	not serious	none	361	335	-	SMD **0.66 SD lower** (0.84 lower to 0.47 lower)	**⊕⊕◯◯** **Low ^l,m^**	Critical
**Low risk of bias**												
7	Randomised trials	not serious	not serious	serious ^n^	not serious	none	256	241	-	SMD **0.56 SD lower** (0.74 lower to 0.38 lower)	**⊕⊕⊕◯** **Moderate ^n^**	Important
**Moderate risk of bias**												
7	Randomised trials	not serious	not serious	serious ^o^	serious ^p^	none	166	161	-	SMD **0.56 SD lower** (0.88 lower to 0.25 lower)	**⊕⊕◯◯** **Low ^o,p^**	Important
**High risk of bias**												
5	Randomised/non-randomised trials	very serious ^q^	not serious	serious ^r^	serious ^s^	none	155	151	-	SMD **0.69 SD lower** (0.98 lower to 0.41 lower)	**⊕◯◯◯** **Very low ^q,r,s^**	Important
**Osteoporotic subjects**												
6	Randomised/non-randomised trials	serious ^t^	not serious	serious ^u^	serious ^v^	none	187	165	-	SMD **0.61 SD lower** (0.92 lower to 0.3 lower)	**⊕◯◯◯** **Very low ^t,u,v^**	Critical
**Subjects without osteoporosis**												
6	Randomised/non-randomised trials	serious ^w^	not serious	serious ^x^	serious ^y^	none	174	170	-	SMD **0.69 SD lower** (0.91 lower to 0.47 lower)	**⊕◯◯◯** **Very low ^w,x,y^**	Critical
**Control group without treatment**												
10	Randomised/non-randomised trials	serious ^z^	not serious	serious ^aa^	not serious	none	320	272	-	SMD **0.73 SD lower** (0.93 lower to 0.54 lower)	**⊕⊕◯◯** **Low ^aa,z^**	Important

## Data Availability

Any personal or patient data are unavailable due to privacy or ethical restrictions. All other data are available from the corresponding author upon reasonable request.

## Abbreviations

The following abbreviations are used in this manuscript:

CI	Confidence Interval
SMD	Standardized Mean Difference
GRADE	Grading of Recommendations, Assessment, Development, and Evaluation
IAC	Isometric Axial Compression
MINORS	Methodological Index for Non-Randomized Studies
NASM	The National Academy of Sports Medicine
PICOS	Population, Intervention, Comparison, Outcome, Study Design
PRISMA	Preferred Reporting Items for Systematic Reviews and Meta-Analyses
PROSPERO	International Prospective Register of Systematic Reviews

## References

[1] Ball J.M., Cagle P., Johnson B.E., Lucasey C., Lukert B. (2009). Spinal extension exercises prevent natural progression of kyphosis. Osteoporos. Int..

[2] Fon G.T., Pitt M.J., Thies A.C. (1980). Normal Kyphosis: Subjects. AJR.

[3] Roghani T., Zavieh M.K., Manshadi F.D., King N., Katzman W. (2018). Age-related hyperkyphosis: Update of its potential causes and clinical impacts—Narrative review Tayebeh. HHS Public Access.

[4] Katzman W.B., Wanek L., Shepherd J.A., Sellmeyer D.E. (2010). Age-Related Hyperkyphosis: Its Causes, Consequences, and Management. J. Orthop. Sport Phys. Ther..

[5] Vaughn D.W., Brown E.W. (2007). The influence of an in-home based therapeutic exercise program on thoracic kyphosis angles. J. Back Musculoskelet. Rehabil..

[6] Kado D.M., Prenovost K., Crandall C. (2007). Narrative review: Hyperkyphosis in older persons. Ann. Intern. Med..

[7] Yaman O., Dalbayrak S. (2014). Kyphosis and review of the literature. Turk. Neurosurg..

[8] Yau M.S., Lam Y.W., Cheung W.H., Kung A.W.C. (2016). Heritability of Thoracic Spine Curvature and Genetic Correlations with Other Spine Traits: The Framingham Study. J. Bone Miner. Res..

[9] Kamali F., Shirazi S.A., Ebrahimi S., Mirshamsi M., Ghanbari A. (2016). Comparison of manual therapy and exercise therapy for postural hyperkyphosis: A randomized clinical trial. Physiother. Theory Pract..

[10] Leech J.A., Dulberg C., Kellle S., Pattee L., Gay J. (1989). Relationship of Lung Function to Severity of Osteoporosis in Women. Ann. Intern. Med..

[11] Ryan S.D., Fried L.P. (1997). The Impact of Kyphosis on Daily Functioning. J. Gerontol. A Biol. Sci. Med. Sci..

[12] Takahashi T., Ishida K., Hirose D. (2005). Trunk deformity is associated with a reduction in outdoor activities of daily living and life satisfaction in community-dwelling older people. Osteoporos. Int..

[13] Farrokhi M.R., Alibai E., Maghami Z. (2011). Randomized controlled trial of percutaneous vertebroplasty versus optimal medical management for the relief of pain and disability in acute osteoporotic vertebral compression fractures. J. Neurosurg. Spine.

[14] Bautmans I., Van Arken J., Van Mackelenberg M., Mets T. (2010). Rehabilitation using manual mobilization for thoracic kyphosis in elderly postmenopausal patients with osteoporosis. J. Rehabil. Med..

[15] Bennell K.L., Matthews B., Greig A., Briggs A., Kelly A., Sherburn M., Larsen J., Wark J. (2010). Effects of an exercise and manual therapy program on physical impairments, function and quality-of-life in people with osteoporotic vertebral fracture: A randomised, single-blind controlled pilot trial. BMC Musculoskelet. Disord..

[16] Perriman D.M., Carty C.P., O’Leary S.P., McMeeken J.M. (2012). Thoracic Hyperkyphosis: A Survey of Australian Physiotherapists. Physiother. Res. Int..

[17] Pfeifer M., Begerow B., Minne H.W. (2004). Effects of a New Spinal Orthosis on Posture, Trunk Strength, and Quality of Life in Women with Postmenopausal Osteoporosis. Am. J. Phys. Med. Rehabil..

[18] Keller T.S., Harrison D.E., Colloca C.J., Harrison D.D., Janik T.J. (2003). Prediction of Osteoporotic Spinal Deformity. Spine.

[19] Hinman M.R. (2004). Comparison of thoracic kyphosis and postural stiffness in younger and older women. J. Geriatr. Phys. Ther..

[20] Katzman W.B., Robison J., Shardell M., Harris T.B., Ferrucci L., Simonsick E.M., Studenski S.A., Xue Q.L., Fried L.P. (2007). Changes in Flexed Posture, Musculoskeletal Impairments, and Physical Performance After Group Exercise in Community-Dwelling Older Women. Phys. Ther..

[21] Katzman W.B., Vittinghoff E., Kado D.M., Schafer A.L., Wong S.S., Gladin A., Lane N.E. (2016). Study of Hyperkyphosis, Exercise and Function (SHEAF) Protocol of a Randomized Controlled Trial of Multimodal Spine-Strengthening Exercise in Older Adults with Hyperkyphosis. Phys. Ther..

[22] Bansal S., Katzman W.B., Giangregorio L.M. (2014). Exercise for improving age-related hyperkyphotic posture: A systematic review. Arch. Phys. Med. Rehabil..

[23] González-Gálvez N., Gea-García G.M., Marcos-Pardo P.J. (2019). Effects of exercise programs on kyphosis and lordosis angle: A systematic review and meta-analysis. PLoS ONE.

[24] Jenkins H.J., Downie A.S., Fernandez M., Hancock M.J. (2021). Decreasing thoracic hyperkyphosis—Which treatments are most effective? A systematic literature review and meta-analysis. Musculoskelet. Sci. Pract..

[25] Ponzano M., Tibert N., Bansal S., Katzman W., Giangregorio L. (2021). Exercise for improving age-related hyperkyphosis: A systematic review and meta-analysis with GRADE assessment. Arch. Osteoporos..

[26] Dimitrijević V., Jovanović M., Stojanović M., Savić J., Milovanović I., Jovanović A. (2021). Effects of corrective exercises on kyphotic angle reduction: A systematic review and meta-analysis. Med. Pregl..

[27] Page M.J., McKenzie J.E., Bossuyt P.M., Boutron I., Hoffmann T.C., Mulrow C.D., Shamseer L., Tetzlaff J.M., Akl E.A., Brennan S.E. (2021). The PRISMA 2020 statement: An updated guideline for reporting systematic reviews. Int. J. Surg..

[28] Higgins J.P.T., Li T., Deeks J.J. (2019). Choosing effect measures and computing estimates of effect. Cochrane Handbook for Systematic Reviews of Interventions.

[29] Slim K., Nini E., Forestier D., Kwiatkowski F., Panis Y., Chauvenet M. (2003). Methodological index for non-randomized studies (MINORS): Development and validation of a new instrument. ANZ J. Surg..

[30] Balduzzi S., Rücker G., Schwarzer G. (2019). How to perform a meta-analysis with R: A practical tutorial. Evid. Based. Ment. Health.

[31] Cohen J. (1988). Statistical Power Analysis for the Behavioral Sciences.

[32] Higgins J.P.T., Thompson S.G. (2002). Quantifying heterogeneity in a meta-analysis. Stat. Med..

[33] Egger M., Davey Smith G., Schneider M., Minder C. (1997). Bias in meta-analysis detected by a simple, graphical test. BMJ.

[34] Guyatt G., Oxman A.D., Vist G.E., Kunz R., Falck-Ytter Y., Glasziou P., Liberati A., Schünemann H.J. (2011). GRADE guidelines: 1. Introduction—GRADE evidence profiles and summary of findings tables. J. Clin. Epidemiol..

[35] Ryan R., Hill S. (2016). How to GRADE the Quality of the Evidence. http://cccrg.cochrane.org/author-resources.

[36] Moustafa I.M., Shousha T.M., Walton L.M., Raigangar V., Harrison D.E. (2022). Reduction of Thoracic Hyper-Kyphosis Improves Short and Long Term Outcomes in Patients with Chronic Nonspecific Neck Pain: A Randomized Controlled Trial. J. Clin. Med..

[37] Seidi F., Rajabi R., Ebrahimi I., Alizadeh M.H., Minoonejad H. (2014). The efficiency of corrective exercise interventions on thoracic hyper-kyphosis angle. J. Back Musculoskelet. Rehabil..

[38] Harding A.T., Harrison D.E., Colloca C.J., Harrison D.D., Janik T.J. (2021). Exploring thoracic kyphosis and incident fracture from vertebral morphology with high-intensity exercise in middle-aged and older men with osteopenia and osteoporosis: A secondary analysis of the LIFTMOR-M trial. Osteoporos. Int..

[39] Çergel Y., Topuz O., Alkan H., Sarsan A., Akkoyunlu N.S. (2019). The effects of short-term back extensor strength training in postmenopausal osteoporotic women with vertebral fractures: Comparison of supervised and home exercise program. Arch. Osteoporos..

[40] Katzman W.B., Vittinghoff E., Kado D.M., Schafer A.L., Wong S.S., Gladin A., Lane N.E. (2017). Targeted spine strengthening exercise and posture training program to reduce hyperkyphosis in older adults: Results from the SHEAF randomized controlled trial. Osteoporos. Int..

[41] Bezalel T., Carmeli E., Levi D., Kalichman L. (2019). The effect of Schroth therapy on thoracic kyphotic curve and quality of life in Scheuermann’s patients: A randomized controlled trial. Asian Spine J..

[42] Elgendy M.H., Mohamed S.R. (2020). Effect of Mulligan Sustained Natural Apophyseal Glides on Thoracic Cobb Angle in Subjects with Thoracic Kyphosis. Egypt. J. Appl. Sci..

[43] Watson S.L., Weeks B.K., Weis L.J., Beck B.R. (2019). High-intensity exercise did not cause vertebral fractures and improves thoracic kyphosis in postmenopausal women with low to very low bone mass: The LIFTMOR trial. Osteoporos. Int..

[44] Feng Q., Wang M., Zhang Y., Zhou Y. (2018). The effect of a corrective functional exercise program on postural thoracic kyphosis in teenagers: A randomized controlled trial. Clin. Rehabil..

[45] Jabbar K.M., Gandomi F. (2021). The comparison of two corrective exercise approaches for hyperkyphosis and forward head posture: A quasi-experimental study. J. Back Musculoskelet. Rehabil..

[46] Yazici A.G., Mohammadi M. (2017). The effect of corrective exercises on the thoracic kyphosis and lumbar lordosis of boy students. Turkish J. Sport Exerc..

[47] Greendale G.A., Huang M.H., Karlamangla A.S., Seeger L., Crawford S. (2009). Yoga decreases kyphosis in senior women and men with adult-onset hyperkyphosis: Results of a randomized controlled trial. J. Am. Geriatr. Soc..

[48] Bulut D., Dilek B., Kilinc A., Ellidokuz H., Oncel S. (2019). An investigation into the effects of kinesiotaping for posture correction on kyphosis angle, pain, and balance in patients with postmenopausal osteoporosis-associated thoracic kyphosis. Arch. Osteoporos..

[49] Jang H.J., Kim M.J., Kim S.Y. (2015). Effect of thorax correction exercises on flexed posture and chest function in older women with age-related hyperkyphosis. J. Phys. Ther. Sci..

[50] Jang H.J., Hughes L.C., Oh D.W., Kim S.Y. (2017). Effects of Corrective Exercise for Thoracic Hyperkyphosis on Posture, Balance, and Well-Being in Older Women: A Double-Blind, Group-Matched Design. J. Geriatr. Phys. Ther..

[51] Navega M.T., Furlanetto M.G., Lorenzo D.M., Morcelli M.H., Tozim B.M. (2016). Effect of the Mat Pilates method on postural balance and thoracic hyperkyphosis among elderly women: A randomized controlled trial. Rev. Bras. Geriatr. Gerontol..

[52] Naderi A., Rezvani M.H., Shaabani F., Bagheri S. (2019). Effect of Kyphosis Exercises on Physical Function, Postural Control and Quality of Life in Elderly Men with Hyperkyphosis. SALMAND-Iranian J. Ageing.

[53] Furukawa T.A., Barbui C., Cipriani A., Brambilla P., Watanabe N. (2006). Imputing missing standard deviations in meta-analyses can provide accurate results. J. Clin. Epidemiol..

[54] Hozo S.P., Djulbegovic B., Hozo I. (2005). Estimating the mean and variance from the median, range, and the size of a sample. BMC Med. Res. Methodol..

